# Echocardiographic Features and Clinical Outcomes of Functional vs. Anatomical Pulmonary Atresia with Intact Ventricular Septum in Neonates

**DOI:** 10.3390/jcdd13020095

**Published:** 2026-02-15

**Authors:** Yalun Qu, Shuang Yang, Yuefeng Cao, Jiachen Li, Zhongyi Han, Dong Wang, Yao Yang, Yongtao Wu, Qiang Wang

**Affiliations:** Beijing Anzhen Hospital of Capital Medical University, Beijing 100029, China; yldr0417@163.com (Y.Q.); 15901103307@126.com (S.Y.); caoyuefeng1220@163.com (Y.C.); lijiachen0915@126.com (J.L.); zhongyihan09@163.com (Z.H.); www2000_83@sina.com (D.W.); cardiac_surgeon@126.com (Y.Y.); wuyt957@126.com (Y.W.)

**Keywords:** functional pulmonary atresia, pulmonary atresia with intact ventricular septum, echocardiography, neonate, differential diagnosis

## Abstract

(1) Background: Functional pulmonary atresia (FPA) and pulmonary atresia with intact ventricular septum (PA/IVS) are rare neonatal congenital heart diseases with similar early clinical manifestations but distinct pathophysiology and treatment strategies, making early and accurate differentiation clinically important. (2) Methods: This single-center retrospective study included 43 neonates diagnosed with FPA (*n* = 12) or PA/IVS (*n* = 31) between December 2016 and March 2025. Echocardiographic parameters and clinical data were compared between groups, and receiver operating characteristic curve analysis was performed to evaluate the usefulness of selected echocardiographic indices for differentiation in clinical practice. (3) Results: Compared with PA/IVS, neonates with FPA exhibited significantly larger right atrial area, relatively better preserved right ventricular development, larger patent ductus arteriosus diameter, and lower peak tricuspid regurgitation velocity. Several parameters, including right atrial area and the right-to-left ventricular ratio, demonstrated strong between-group discrimination in this cohort. Clinically, most FPA neonates were managed conservatively with favorable outcomes, whereas PA/IVS neonates required surgical intervention and experienced higher perioperative mortality. (4) Conclusions: FPA and PA/IVS differ significantly in right heart morphology, hemodynamic characteristics, and management strategies. A comprehensive multi-parameter echocardiographic evaluation demonstrated discriminatory ability in this cohort, facilitating appropriate treatment decisions and potentially helping to avoid unnecessary high-risk surgical interventions.

## 1. Introduction

Pulmonary atresia with intact ventricular septum (PA/IVS) is a rare cyanotic congenital heart disease with significant anatomical variations. Most cases of PA/IVS present with membranous pulmonary valve (PV) atresia, while some exhibit muscular atresia. The condition is characterized by significant morphological heterogeneity, often associated with varying degrees of right ventricular (RV) hypoplasia and may involve right-ventricular-dependent coronary circulation (RVDCC), pulmonary atresia (PA), and associated tricuspid valve (TV) abnormalities [1,2,3]. Children with PA/IVS typically experience severe cyanosis and have a high mortality rate without intervention or treatment. In contrast, functional pulmonary atresia (FPA) refers to a phenomenon where the pulmonary valve remains structurally normal but is unable to open due to an imbalance in pressure between the RV and pulmonary artery [4]. This rare condition is typically associated with increased pulmonary vascular resistance (PVR) during the perinatal period [5], Ebstein’s anomaly [6,7,8], tricuspid valve regurgitation (TVR) [9], Uhl’s anomaly [10], neonatal Marfan syndrome [4,11], fetal cardiomyopathy, and right heart dysfunction. There are also reports of FPA without any apparent intracardiac structural anomalies [12,13]. When pulmonary vascular resistance decreases to a certain level or when the RV generates sufficient pressure, the pulmonary valve opens [14]. Differentiating functional from structural pulmonary atresia is crucial, as this distinction has therapeutic implications and can help avoid unnecessary high-risk surgery for FPA patients. Therefore, we conducted a single-center retrospective study to summarize the echocardiographic features of FPA and PA/IVS in neonates, providing a basis for the differential diagnosis of PA/IVS and FPA.

## 2. Materials and Methods

### 2.1. Echocardiography and Echocardiographic Measurements

Patients were classified as having functional pulmonary atresia (FPA) or pulmonary atresia with intact ventricular septum (PA/IVS) based on the final clinical diagnosis made by experienced pediatric cardiologists and cardiac surgeons. The diagnosis was established through comprehensive assessment, including clinical presentation, echocardiographic findings, intraoperative observations when available, and postnatal clinical course.

Echocardiographic examinations were performed immediately upon hospital admission as part of routine clinical evaluation. All studies were conducted by experienced pediatric cardiology echocardiographers using a Philips EPIQ 7C ultrasound system (Philips Medical Systems, Andover, MA, USA) equipped with high-frequency neonatal transducers (S8-3 or S12-4), selected according to patient size and acoustic window to optimize image quality. Measurements were obtained in real time during the original examinations.

The echocardiographic parameters collected included right atrial diameter and area, right ventricular free wall thickness, right ventricular outflow tract diameter, right ventricular to left ventricular dimension ratio (RV/LV ratio), pulmonary valve annular diameter and Z-score, main pulmonary artery and branch pulmonary artery diameters, mitral and tricuspid valve annular diameters and corresponding Z-scores, peak tricuspid regurgitation velocity (TRPV), foramen ovale/atrial septal defect size, and patent ductus arteriosus (PDA) diameter. TRPV was originally recorded in cm/s in the echocardiographic reports and converted to m/s for descriptive comparison when appropriate.

Echocardiographic parameters were not used as predefined criteria for group assignment but were retrospectively analyzed to describe and compare phenotypic characteristics between the clinically diagnosed groups. Because echocardiographic examinations were performed as part of routine clinical care, formal blinding to the final diagnosis was not feasible, and inter- or intra-observer variability analyses were not performed in this retrospective study.

### 2.2. Medical Treatment

During the study period, treatment strategies were determined according to institutional practice and underlying pathophysiology. Patients with functional pulmonary atresia were generally managed conservatively. Prostaglandin E1 (PGE1) was administered to maintain ductus arteriosus patency and reduce pulmonary arterial pressure and was discontinued once pulmonary valve opening and antegrade pulmonary blood flow were restored in order to avoid excessive ductal flow.

In patients with pulmonary atresia with intact ventricular septum, surgical management was planned based on the degree of right ventricular development, aiming for early biventricular repair when feasible. Surgical strategies included enlargement of the right ventricular outflow tract and main pulmonary artery, partial preservation of the patent ductus arteriosus (approximately 3 mm), and preservation of the foramen ovale when indicated. Hybrid procedures involving pulmonary valvuloplasty with balloon dilation via median sternotomy were applied according to institutional protocols, and in selected cases, a modified Blalock–Taussig shunt was used for palliative augmentation of pulmonary blood flow.

Transcatheter pulmonary valvuloplasty has been reported as an alternative option in the literature; however, this percutaneous approach was not routinely performed at our center during the study period and was therefore not included in the present study.

### 2.3. Clinical Outcomes

Clinical outcomes assessed in this study included in-hospital neonatal mortality. All outcome data were retrospectively extracted from institutional electronic medical records maintained by professional clinical staff.

### 2.4. Statistical Analysis

Continuous variables were expressed as mean ± standard deviation. For normally distributed data, comparisons between groups were performed using the independent samples t-test. Non-normally distributed data were analyzed using the Mann–Whitney U test. Categorical variables were expressed as counts and percentages and compared using the chi-square test. A *p*-value < 0.05 was considered statistically significant.

Receiver operating characteristic (ROC) curve analysis was performed to evaluate the discriminatory performance of selected echocardiographic parameters in differentiating functional pulmonary atresia from pulmonary atresia with intact ventricular septum. The area under the curve (AUC) was calculated for each parameter.

## 3. Results

### 3.1. Sample Characteristics and Treatment Modalities

A total of 43 neonates were included in the final analysis, comprising 12 with functional pulmonary atresia (FPA) and 31 with pulmonary atresia with intact ventricular septum (PA/IVS). There were no significant differences between the two groups with respect to sex, admission SpO_2_, or in-hospital mortality, whereas significant differences were observed in birth weight, gestational age, and treatment distribution.

**Figure 1:**
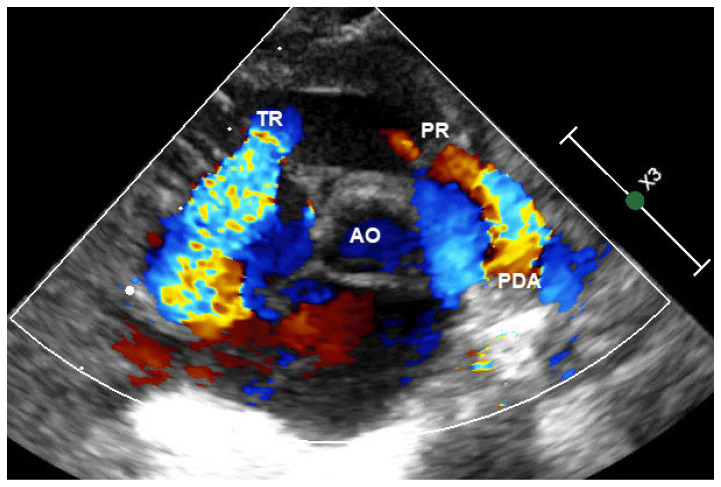
Echocardiographic findings in a neonate with functional pulmonary atresia at admission. Color Doppler imaging demonstrates a thick patent ductus arteriosus (PDA) with turbulent pulmonary arterial flow, while no antegrade blood flow across the pulmonary valve (PV) is observed. Abbreviations: PDA, patent ductus arteriosus; PV, pulmonary valve.

**Figure 2:**
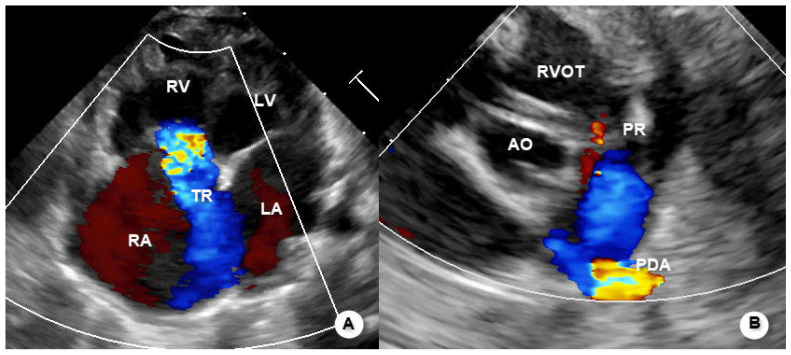
Representative echocardiographic images of functional pulmonary atresia. (**A**) Apical four-chamber view demonstrating enlargement of the right atrium (RA) and right ventricle (RV) with severe tricuspid regurgitation (TR). (**B**) Parasternal short-axis view showing absence of antegrade pulmonary valve flow, with minimal pulmonary regurgitation (PR); pulmonary arterial blood flow is supplied predominantly via the patent ductus arteriosus (PDA). Abbreviations: RA, right atrium; RV, right ventricle; LA, left atrium; LV, left ventricle; RVOT, right ventricular outflow tract; AO, aorta; TR, tricuspid regurgitation; PR, pulmonary regurgitation; PDA, patent ductus arteriosus.

**Figure 3:**
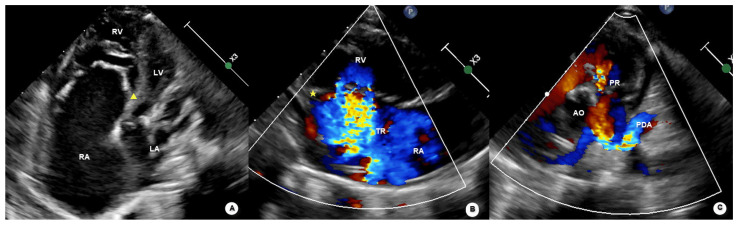
Echocardiographic features of functional pulmonary atresia demonstrating right heart enlargement and ductal-dependent pulmonary blood flow. (**A**) Apical four-chamber view showing enlargement of the right atrium (RA) and right ventricle (RV), with leftward displacement of the interventricular septum (arrow). (**B**) Color Doppler imaging demonstrating severe tricuspid regurgitation (TR) associated with right heart dilation. (**C**) Color Doppler imaging of the pulmonary artery region showing absence of antegrade pulmonary valve flow, with pulmonary blood flow supplied predominantly via a tortuous patent ductus arteriosus (PDA) and minimal pulmonary regurgitation (PR). Abbreviations: RA, right atrium; RV, right ventricle; LA, left atrium; LV, left ventricle; AO, aorta; TR, tricuspid regurgitation; PR, pulmonary regurgitation; PDA, patent ductus arteriosus.

**Figure 4:**
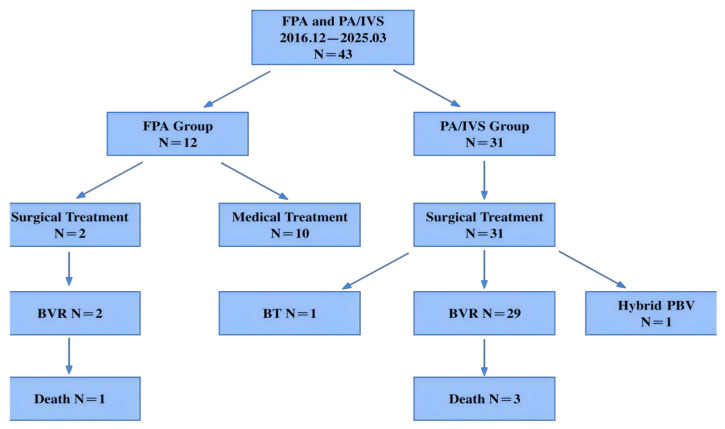
Flow diagram illustrating patient classification, treatment distribution, and in-hospital outcomes in neonates with functional pulmonary atresia (FPA) and pulmonary atresia with intact ventricular septum (PA/IVS) during the study period (December 2016 to March 2025).

As illustrated in the flow diagram (Figure 1, Figure 2, Figure 3 and Figure 4), initial preoperative assessment classified 10 patients as FPA and 33 as PA/IVS. During subsequent surgical exploration or clinical course, two patients initially diagnosed as PA/IVS were found to have an intact pulmonary valve without adhesions or membranous atresia and were reclassified as FPA. Surgical intervention in these cases was prompted by specific clinical indications, including malignant supraventricular tachycardia associated with marked right atrial enlargement and a large hemodynamically significant patent ductus arteriosus.

Regarding treatment distribution, most FPA neonates were managed conservatively (10/12). Among the two FPA patients who underwent surgical intervention, one received a modified Blalock–Taussig shunt and one underwent a hybrid procedure with pulmonary valvuloplasty via median sternotomy. In contrast, all PA/IVS neonates underwent surgical treatment (31/31), including biventricular repair in the majority and hybrid pulmonary valvuloplasty in one case. One FPA neonate died postoperatively due to necrotizing enterocolitis and septic shock, and three PA/IVS neonates died during the postoperative period (Table 1). Treatment distribution and in-hospital outcomes are summarized in the flow diagram shown in Figure 4.

### 3.2. Echocardiographic Features

Representative echocardiographic images illustrating the typical morphological and hemodynamic features of functional pulmonary atresia are shown in Figure 1, Figure 2 and Figure 3.

**Figure 5:**
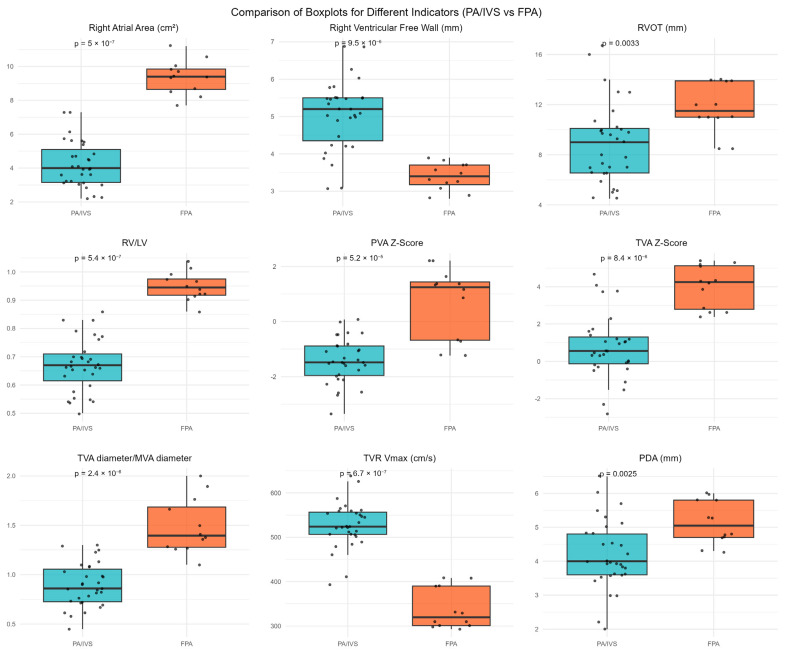
Box-and-whisker plots comparing key echocardiographic parameters between neonates with pulmonary atresia with intact ventricular septum (PA/IVS) and functional pulmonary atresia (FPA). The plots illustrate differences in right atrial area, right ventricular free wall thickness, right ventricular outflow tract (RVOT) diameter, right ventricular to left ventricular (RV/LV) ratio, pulmonary valve annulus (PVA) Z-score, tricuspid valve annulus (TVA) Z-score, tricuspid-to-mitral annular diameter ratio, peak tricuspid regurgitation velocity (TR Vmax), and patent ductus arteriosus (PDA) diameter. Boxes represent the median and interquartile range, whiskers indicate the full range, and individual dots denote individual patients. *p*-values were calculated using the Mann–Whitney U test.

Right atrial area (RAA) in the FPA group was significantly larger than in the PA/IVS group (*p* < 0.001). Forty-eight percent of PA/IVS cases had RV hypoplasia, while no RV hypoplasia was found in the FPA group (*p* = 0.003). RV free wall thickness was significantly greater in PA/IVS cases than in FPA cases (*p* < 0.001). Conversely, the RV/LV ratio was significantly larger in the FPA group (*p* < 0.001). Ten FPA cases had pulmonary valve regurgitation (PVR), while two did not. All PA/IVS cases had no pulmonary valve regurgitation (*p* < 0.001). Pulmonary valve annulus diameter and Z-score were significantly higher in the FPA group compared to the PA/IVS group (*p* < 0.001). Tricuspid valve annulus diameter (TVAD), tricuspid valve Z-score (TV Z), and the ratio of tricuspid valve annulus to mitral valve annulus (TV/MV ratio) were significantly greater in the FPA group compared to PA/IVS cases (*p* < 0.001). However, peak tricuspid regurgitation velocity (TRPV, m/s; converted from cm/s) was significantly lower in the FPA group compared to the PA/IVS group (*p* < 0.001). Additionally, PDA diameter was larger in the FPA group (*p* = 0.003). See Table 2 and Figure 5.

### 3.3. ROC Curve Analysis

After excluding parameters with mathematical interdependence, the following echocardiographic variables were selected for receiver operating characteristic (ROC) curve analysis: right atrial area, right ventricular (RV) free wall thickness, RV outflow tract diameter, RV/left ventricular (LV) ratio, pulmonary valve annulus Z-score, tricuspid valve annulus Z-score, tricuspid-to-mitral valve (TV/MV) ratio, peak tricuspid regurgitation velocity (TRPV), and patent ductus arteriosus (PDA) diameter.

ROC analysis demonstrated excellent discriminatory performance for several parameters. Right atrial area showed perfect discrimination, with an optimal cut-off value of 7.5 (AUC = 1.00, 95% CI: 1.00–1.00; *p* < 0.001), yielding a sensitivity and specificity of 100%. RV free wall thickness had an optimal cut-off value of 3.95 (AUC = 0.94, 95% CI: 0.866–1.00; *p* < 0.001), with 100% sensitivity and 87.1% specificity. The optimal cut-off value for RV outflow tract diameter was 10.85 (AUC = 0.793, 95% CI: 0.654–0.932; *p* = 0.003), with a sensitivity of 83.3% and specificity of 80.6%.

The RV/LV ratio also demonstrated near-perfect discrimination, with a cut-off value of 0.84 (AUC = 0.999, 95% CI: 0.995–1.00; *p* < 0.001), corresponding to a sensitivity of 100% and specificity of 96.8%. The pulmonary valve annulus Z-score showed an optimal cut-off value of 0.46 (AUC = 0.90, 95% CI: 0.804–1.00; *p* < 0.001), with a sensitivity of 66.7% and specificity of 100%. Similarly, the tricuspid valve annulus Z-score demonstrated strong discriminatory ability, with a cut-off value of 2.33 (AUC = 0.94, 95% CI: 0.880–1.00; *p* < 0.001), yielding a sensitivity of 100% and specificity of 87.1%.

The TV/MV ratio exhibited excellent performance, with an optimal cut-off value of 1.25 (AUC = 0.96, 95% CI: 0.926–1.00; *p* < 0.001), corresponding to a sensitivity of 91.7% and specificity of 93.5%. TRPV demonstrated near-perfect discrimination, with a cut-off value of 409.5 cm/s (corresponding to 4.10 m/s) (AUC = 0.995, 95% CI: 0.982–1.00), yielding a sensitivity of 100% and specificity of 96.8%. PDA diameter showed moderate discriminatory performance, with an optimal cut-off value of 4.25 (AUC = 0.801, 95% CI: 0.672–0.93; *p* = 0.002), corresponding to a sensitivity of 100% and specificity of 61.3%.

**Figure 6:**
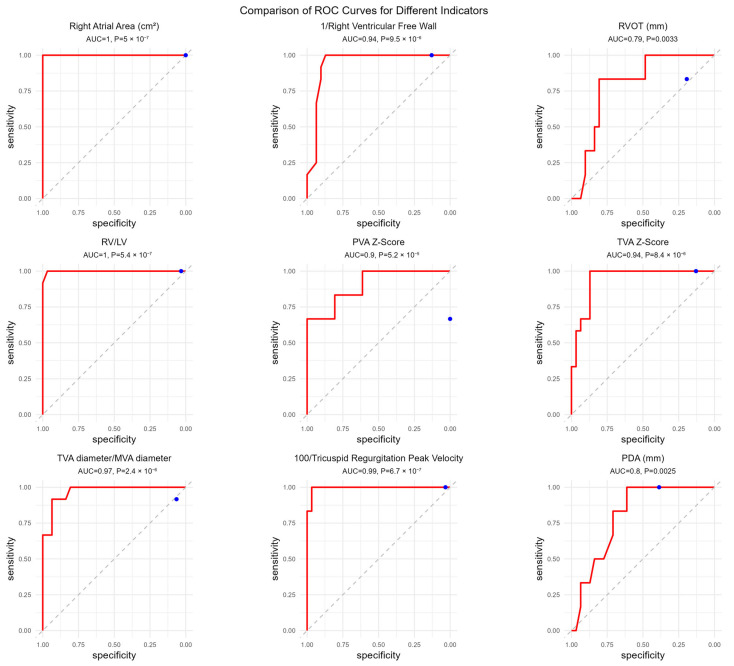
Receiver operating characteristic (ROC) curves illustrating the discriminatory performance of selected echocardiographic parameters for differentiating functional pulmonary atresia (FPA) from pulmonary atresia with intact ventricular septum (PA/IVS) within the present cohort. ROC analyses were performed for right atrial area, right ventricular free wall thickness, right ventricular outflow tract (RVOT) diameter, right ventricular to left ventricular (RV/LV) ratio, pulmonary valve annulus (PVA) Z-score, tricuspid valve annulus (TVA) Z-score, tricuspid-to-mitral annular diameter ratio, peak tricuspid regurgitation velocity, and patent ductus arteriosus (PDA) diameter. The area under the curve (AUC) and corresponding *p*-values are displayed for each parameter. These analyses reflect cohort-specific discriminatory performance rather than validated independent diagnostic prediction models.

Detailed ROC curves and diagnostic performance metrics are presented in Table 3 and Figure 6. To facilitate visualization with positive ROC curves, reciprocal transformation was applied to RV free wall thickness and peak tricuspid regurgitation velocity.

The diagram shows the final clinical classification of 43 neonates, subsequent allocation to medical or surgical treatment, distribution of surgical procedures (biventricular repair, modified Blalock–Taussig shunt, and hybrid pulmonary valvuloplasty), and in-hospital mortality in each group.

## 4. Discussion

This study systematically reviewed and compared 43 neonates diagnosed with functional pulmonary atresia (FPA) and pulmonary atresia with intact ventricular septum (PA/IVS) treated at our center. To our knowledge, published studies focusing on the echocardiographic differentiation between FPA and PA/IVS are limited and mostly consist of small case series. In the present cohort, clear differences were observed between the two groups in right heart morphology, hemodynamic characteristics, and treatment strategies, and several echocardiographic parameters showed clear between-group discriminatory ability in this cohort.

Special attention should be given to the two FPA cases that were initially misclassified as PA/IVS before surgery. Intraoperative findings confirmed that their pulmonary valves were structurally intact without membranous atresia or fusion, and the final diagnosis was revised to FPA. These cases underscore that the diagnosis of structural pulmonary atresia should not be based solely on the absence of pulmonary valve regurgitation. Overreliance on a single echocardiographic sign may lead to misclassification and unnecessary high-risk surgical intervention. Instead, a comprehensive evaluation incorporating right heart morphology (including right atrial and ventricular size), tricuspid valve characteristics, and the status of the patent ductus arteriosus (PDA) is important for appropriate differentiation.

Our comparative analysis demonstrated notable differences in cardiac morphology, hemodynamics, and clinical management between FPA and PA/IVS. Neonates with FPA exhibited enlarged right atria, relatively well-developed right ventricles, larger pulmonary valve annulus diameters and Z-scores, and wider PDA diameters compared with those with PA/IVS. In contrast, PA/IVS patients more frequently showed smaller right heart structures, thicker right ventricular free walls, and higher tricuspid regurgitation velocities. Receiver operating characteristic (ROC) curve analysis indicated that parameters such as right atrial area, right ventricular free wall thickness, RV/LV ratio, tricuspid regurgitation peak velocity, and the tricuspid-to-mitral annular ratio demonstrated clear discrimination between the two groups. This finding should be interpreted cautiously, as it reflects cohort-specific separation rather than validated diagnostic thresholds. Clinically, most FPA neonates were managed conservatively with favorable short-term outcomes, whereas all PA/IVS patients required surgical intervention and experienced slightly higher in-hospital mortality.

From a pathophysiological perspective, functional pulmonary atresia occurs when pulmonary artery systolic pressure exceeds right ventricular systolic pressure, preventing opening of an anatomically intact but narrowed pulmonary valve. This hemodynamic condition may arise in the presence of pulmonary hypertension, right ventricular systolic or diastolic dysfunction, or significant tricuspid valve dysfunction. Clarifying this mechanism helps explain why functional obstruction can occur despite preserved pulmonary valve anatomy and underscores the importance of integrating hemodynamic context into echocardiographic assessment.

In this study, right atrial enlargement was a prominent feature among neonates with FPA. Two potential mechanisms may explain this finding. First, FPA patients typically have a relatively compliant and well-developed right ventricle, allowing larger tricuspid regurgitant volumes and increased right atrial volume load. In contrast, PA/IVS patients often have hypoplastic right ventricles; although tricuspid regurgitation velocity may be higher, the overall regurgitant volume is usually limited. Second, some FPA cases were associated with Ebstein’s anomaly or tricuspid valve dysplasia. In these conditions, apical displacement of the tricuspid valve and severe tricuspid regurgitation result in marked right atrial enlargement and reduced effective right ventricular systolic pressure. This mechanism represents one of the more common and clinically relevant contributors to functional pulmonary atresia and should be carefully considered during echocardiographic evaluation.

Right ventricular development represents an important distinguishing characteristic between the two diseases. It should be noted, however, that pulmonary atresia with intact ventricular septum encompasses a wide spectrum of right ventricular morphology. Although most cases present with right ventricular hypoplasia and hypertrophy, a subset of patients may exhibit right ventricular dilation and severe tricuspid regurgitation, which can complicate differentiation from functional pulmonary atresia based on morphology alone. PA/IVS is commonly associated with right ventricular hypoplasia and, in severe cases, right ventricular–dependent coronary circulation, whereas FPA typically presents with a thin-walled, compliant, and adequately sized right ventricle. Echocardiographic indices such as right ventricular free wall thickness, RV/LV ratio, tricuspid valve Z-score, and the tricuspid-to-mitral annular ratio reflect these developmental differences. In PA/IVS, increased right ventricular wall thickness and reduced cavity size are frequently associated with diastolic dysfunction and impaired postoperative filling, necessitating continued dependence on ductal flow. Early surgical intervention is therefore often required to support right ventricular growth, and perinatal surgical strategies have been increasingly adopted at our center to promote right heart development at an early stage.

In fetal circulation, both FPA and PA/IVS are characterized by reversed pulmonary perfusion via the ductus arteriosus. After birth, premature ductal constriction may result in severe hypoxemia. In the present study, FPA neonates demonstrated significantly larger PDA diameters than those with PA/IVS, suggesting a greater contribution of ductal flow to pulmonary perfusion. FPA frequently coexists with tricuspid valve dysplasia or Ebstein’s anomaly, conditions in which right ventricular pressure may be insufficient to open an anatomically normal pulmonary valve, resulting in functional atresia. Although tricuspid valve annular Z-scores were higher in FPA cases, this parameter alone appeared to have limited value for differentiation.

Previous reports on FPA have been largely confined to isolated case reports or small series [15,16,17,18,19]. Yeager et al. [20] described echocardiographic features suggestive of FPA, including pulmonary valve regurgitation, a normal or dilated right ventricle and infundibulum, a normal main pulmonary artery diameter, and a morphologically normal ductus arteriosus. Subsequent work by Suzuki et al. [6] emphasized that the absence of pulmonary valve regurgitation does not exclude FPA, consistent with our observations. Our findings further suggest that even in the absence of pulmonary valve regurgitation, the coexistence of a well-developed right ventricle, marked right atrial enlargement, an increased tricuspid-to-mitral annular ratio, and a relatively large PDA may be indicative of FPA. Hiraumi et al. [21] reported that an RV end-diastolic diameter/LV end-diastolic diameter ratio > 0.6 and lower tricuspid regurgitation velocity were more frequently observed in FPA, findings that are in agreement with our results obtained using quantitative analysis in a larger cohort. In addition, right ventricular coronary fistulas, which have been reported to be rare in FPA, were not observed in our study [15].

Several limitations of this study should be acknowledged. First, several echocardiographic features analyzed in this study overlap with elements considered during clinical diagnosis; therefore, between-group comparisons should be interpreted as descriptive phenotypic differences rather than independent diagnostic predictors. Second, this was a single-center retrospective study with a relatively limited sample size, which may restrict the generalizability of the findings. In addition, echocardiographic measurements were obtained as part of routine clinical care, and strict blinding, inter- or intra-observer variability analyses, and comprehensive hemodynamic assessment—including pulmonary artery pressure estimation using tricuspid regurgitation or ductal flow velocities—were not consistently available across all cases. Furthermore, although transcatheter pulmonary valve perforation and balloon valvuloplasty have been increasingly adopted for selected patients with membranous pulmonary atresia [22], such catheter-based interventions were not routinely performed at our center during the study period and were therefore not included in the present analysis.

## 5. Conclusions

This study retrospectively compared the echocardiographic characteristics and clinical features of neonates with functional pulmonary atresia (FPA) and pulmonary atresia with intact ventricular septum (PA/IVS).

The identification of two preoperatively misdiagnosed cases underscores the clinical importance of comprehensive echocardiographic evaluation in real-world practice. The two conditions differ substantially in clinical management, with important implications for therapeutic decision-making in the neonatal period. Accurate recognition of these entities may help guide appropriate clinical management and reduce the risk of unnecessary high-risk interventions in the neonatal period.

## Figures and Tables

**Table 1:** Baseline clinical characteristics and treatment distribution.

Variable	FPA (*n* = 12)	PA/IVS (*n* = 31)	*p*-Value
Sex at birth (% male)	8 (66%)	20 (64%)	0.894
Weight (kg)	2.8 ± 0.38	3.2 ± 0.57	0.009
Gestational Age (weeks)	36.1 ± 1.9	37.5 ± 1.3	0.012
SpO_2_ (%)	82.6 ± 6.6	80.8 ± 10.8	0.591
Medical Treatment	10 (83%)	0 (0%)	<0.001
Surgical Treatment	2 (17%)	31 (100%)	<0.001
ICU Stay (days)	20.2 ± 8.2	19.4 ± 14.3	0.852
Length of Hospital Stay (days)	24.0 ± 7.43	25.1 ± 13.8	0.785
In-hospital Mortality	1 (8%)	3 (9%)	0.892

**Table 2:** Echocardiographic characteristics of the study population.

Variable	FPA (*n* = 12)	PA/IVS (*n* = 31)	*p*-Value
Right Ventricular Hypoplasia	0 (0%)	15 (48%)	0.003
RV Coronary Fistula	0 (0%)	1 (3.2%)	0.529
RVFW (mm)	3.42 ± 0.35	5.05 ± 0.93	<0.001
RVOT (mm)	11.7 ± 1.97	8.91 ± 3.21	0.004
RAA (cm^2^)	9.87 ± 1.41	4.21 ± 1.35	<0.001
RV/LV	0.94 ± 0.05	0.67 ± 0.09	<0.001
LVDd (mm)	22.07 ± 0.80	19.73 ± 3.48	<0.001
LVDs (mm)	13.11 ± 1.79	12.47 ± 2.50	0.211
LVEF (%)	70.67 ± 8.19	69.06 ± 4.85	0.268
Pulmonary Forward Flow	0 (0%)	0 (0%)	1.000
Pulmonary Valve Regurgitation	10 (83%)	0 (0%)	<0.001
PVAD (mm)	8.92 ± 1.39	7.24 ± 0.63	<0.001
PVA Z-Score	0.69 ± 1.28	−1.42 ± 0.80	<0.001
MPA (mm)	9.69 ± 1.40	9.19 ± 1.53	0.332
LPA (mm)	4.48 ± 0.60	4.44 ± 0.43	0.804
RPA (mm)	4.70 ± 0.67	4.58 ± 0.64	0.612
MVAD (mm)	12.35 ± 1.36	13.20 ± 1.44	0.089
TVAD (mm)	18.16 ± 3.15	11.68 ± 2.70	<0.001
TVA Z-Score	4.01 ± 1.14	0.74 ± 1.71	<0.001
TVA Diameter/MVA Diameter	1.48 ± 0.27	0.89 ± 0.22	<0.001
TVR-Vmax (mm/s)	339.08 ± 45.97	526.68 ± 51.28	<0.001
FO/ASD (mm)	4.76 ± 0.51	4.72 ± 0.93	0.856
PDA (mm)	5.15 ± 0.63	4.16 ± 1.01	0.003

RV: Right Ventricle; RVFW: Right Ventricular Free Wall; RVOT: Right Ventricular Outflow Tract; RAA: Right Atrial Area; RV/LV: Right Ventricle/Left Ventricle Ratio; LVDd: Left Ventricular End-Diastolic Diameter; LVDs: Left Ventricular End-Systolic Diameter; LVEF: Left Ventricular Ejection Fraction; PVAD: Pulmonary Valve Annular Diameter; PVA Z-Score: Pulmonary Valve Annular Diameter Z-Score; MPA: Main Pulmonary Artery; LPA: Left Pulmonary Artery; RPA: Right Pulmonary Artery; MVAD: Mitral Valve Annular Diameter; TVAD: Tricuspid Valve Annular Diameter; TVA Z-Score: Tricuspid Valve Annular Diameter Z-Score; TVA Diameter/MVA Diameter: Tricuspid Valve Annular Diameter/Mitral Valve Annular Diameter Ratio; TVR-Vmax: Tricuspid Valve Regurgitation Peak Velocity; FO/ASD: Foramen Ovale/Atrial Septal Defect; PDA: Patent Ductus Arteriosus.

**Table 3:** Receiver operating characteristic (ROC) analysis of echocardiographic variables for differentiating between groups.

Variable	AUC	CI-Lower	CI-Upper	Sensitivity	Specificity	Cutoff	*p*-Value
RAA	1.000	1.000	1.000	1.000	1.000	7.500	<0.001
RV Free Wall	0.940	0.866	1.000	1.000	0.871	3.95	<0.001
RVOT	0.793	0.654	0.932	0.833	0.806	10.850	0.003
RV/LV	0.999	0.995	1.000	1.000	0.968	0.845	<0.001
PVA Z-Score	0.903	0.804	1.000	0.667	1.000	0.465	<0.001
TVA Z-Score	0.944	0.880	1.000	1.000	0.871	2.335	<0.001
TVA Diameter/MVA Diameter	0.969	0.926	1.000	0.917	0.935	1.255	<0.001
TVR-Vmax	0.995	0.982	1.000	1.000	0.968	409.5	<0.001
PDA	0.801	0.672	0.930	1.000	0.613	4.250	0.002

AUC: Area Under the ROC Curve; CI: Confidence Interval.

## Data Availability

The original contributions presented in this study are included in the article. Further inquiries can be directed to the corresponding author.

## Abbreviations

The following abbreviations are used in this manuscript:

AO	Aorta
ASD	Atrial Septal Defect
AUC	Area Under the Curve
BVR	Biventricular Repair
CI	Confidence Interval
FPA	Functional Pulmonary Atresia
FO	Foramen ovale
ICU	Intensive Care Unit
LA	Left Atrium
LV	Left Ventricle
LPA	Left Pulmonary Artery
LVDd	Left Ventricular End-Diastolic Diameter
LVDs	Left Ventricular End-Systolic Diameter
LVEF	Left Ventricular Ejection Fraction
MPA	Main Pulmonary Artery
MV	Mitral Valve
MVAD	Mitral Valve Annular Diameter
NEC	Necrotizing Enterocolitis
PA	Pulmonary Atresia
PA/IVS	Pulmonary Atresia with Intact Ventricular Septum
PDA	Patent Ductus Arteriosus
PGE1	Prostaglandin E1
PR	Pulmonary Regurgitation
PV	Pulmonary Valve
PVAD	Pulmonary Valve Annular Diameter
PVR	Pulmonary Vascular Resistance
RA	Right Atrium
RAA	Right Atrial Area
ROC	Receiver Operating Characteristic
RPA	Right Pulmonary Artery
RV	Right Ventricle
RVDCC	Right Ventricular–Dependent Coronary Circulation
RVFW	Right Ventricular Free Wall
RVOT	Right Ventricular Outflow Tract
SpO_2_	Peripheral Oxygen Saturation
TR	Tricuspid Regurgitation
TRPV	Tricuspid Regurgitation Peak Velocity
TV	Tricuspid Valve
TVAD	Tricuspid Valve Annular Diameter
